# Supplementary material for: Modeling glioblastoma heterogeneity as a dynamic network of cell states
Source: Mol Syst Biol. 2021 Sep 16;17(9):e10105. doi: 10.15252/msb.202010105 (PMC8444284; doi:10.15252/msb.202010105)
Supplement: Supplementary file 6 — Source Data for Figure 5 [file MSB-17-e10105-s004.zip › Figure5A_sourcedata/GSEA_3017/hallmarks_stateF.GseaPreranked.1621934628066/HALLMARK_INFLAMMATORY_RESPONSE.html]

Details for gene set HALLMARK\_INFLAMMATORY\_RESPONSE[GSEA]

|  || Dataset | state33017 |
| Phenotype | NoPhenotypeAvailable |
| Upregulated in class | na\_pos |
| GeneSet | HALLMARK\_INFLAMMATORY\_RESPONSE |
| Enrichment Score (ES) | 0.5661675 |
| Normalized Enrichment Score (NES) | 2.4801326 |
| Nominal p-value | 0.0 |
| FDR q-value | 0.0 |
| FWER p-Value | 0.0 |
Table: GSEA Results Summary

  

Fig 1: Enrichment plot: HALLMARK\_INFLAMMATORY\_RESPONSE      
 Profile of the Running ES Score & Positions of GeneSet Members on the Rank Ordered List

  

| PROBE | GENE SYMBOL | GENE\_TITLE | RANK IN GENE LIST | RANK METRIC SCORE | RUNNING ES | CORE ENRICHMENT || 1 | CDKN1A |  |  | 4 | 2.089 | 0.1219 | Yes |
| 2 | CCL2 |  |  | 7 | 1.774 | 0.2260 | Yes |
| 3 | SLC4A4 |  |  | 40 | 0.980 | 0.2696 | Yes |
| 4 | F3 |  |  | 41 | 0.978 | 0.3275 | Yes |
| 5 | ABCA1 |  |  | 48 | 0.938 | 0.3803 | Yes |
| 6 | BST2 |  |  | 55 | 0.910 | 0.4315 | Yes |
| 7 | SLC1A2 |  |  | 63 | 0.857 | 0.4791 | Yes |
| 8 | BTG2 |  |  | 124 | 0.708 | 0.4941 | Yes |
| 9 | NAMPT |  |  | 168 | 0.634 | 0.5123 | Yes |
| 10 | ATP2B1 |  |  | 170 | 0.632 | 0.5492 | Yes |
| 11 | ATP2A2 |  |  | 210 | 0.582 | 0.5662 | Yes |
| 12 | CSF1 |  |  | 333 | 0.495 | 0.5407 | No |
| 13 | KCNJ2 |  |  | 607 | 0.396 | 0.4416 | No |
| 14 | CHST2 |  |  | 628 | 0.390 | 0.4557 | No |
| 15 | RGS16 |  |  | 691 | 0.373 | 0.4499 | No |
| 16 | AHR |  |  | 942 | 0.319 | 0.3565 | No |
| 17 | ITGB8 |  |  | 970 | 0.314 | 0.3630 | No |
| 18 | HIF1A |  |  | 972 | 0.314 | 0.3811 | No |
| 19 | OSMR |  |  | 1111 | 0.291 | 0.3364 | No |
| 20 | PTPRE |  |  | 1243 | 0.275 | 0.2938 | No |
| 21 | CX3CL1 |  |  | 1273 | 0.272 | 0.2969 | No |
| 22 | KIF1B |  |  | 1276 | 0.271 | 0.3120 | No |
| 23 | PDE4B |  |  | 1404 | 0.256 | 0.2701 | No |
| 24 | ADRM1 |  |  | 1497 | -0.258 | 0.2441 | No |
| 25 | HBEGF |  |  | 1583 | -0.282 | 0.2226 | No |
| 26 | HAS2 |  |  | 1968 | -0.464 | 0.0776 | No |
| 27 | SERPINE1 |  |  | 2161 | -0.843 | 0.0413 | No |
Table: GSEA details [plain text format]

  

Fig 2: HALLMARK\_INFLAMMATORY\_RESPONSE: Random ES distribution      
 Gene set null distribution of ES for **HALLMARK\_INFLAMMATORY\_RESPONSE**
